# Editorial: Synthetic biology and therapeutic applications of transfer RNA

**DOI:** 10.3389/fgene.2024.1468891

**Published:** 2024-08-14

**Authors:** Dieter Söll, Patrick O’Donoghue, Ilka U. Heinemann

**Affiliations:** ^1^ Departments of Molecular Biophysics and Biochemistry, and Chemistry, Yale University, New Haven, CT, United States; ^2^ Department of Biochemistry, The University of Western Ontario, London, ON, Canada; ^3^ Department of Chemistry, The University of Western Ontario, London, ON, Canada; ^4^ Children’s Health Research Institute, London, ON, Canada

**Keywords:** RNA, genetics, synthetic biology, tRNA, therapeutics

## Introduction

In all living cells, the genetic code defines the relationship between nucleic acid sequences in protein coding genes with the sequences of amino acids needed to accurately produce all the proteins encoded in the genome. The aminoacylation of transfer RNAs (tRNAs) by the aminoacyl-tRNA synthetases is the critical step that physically links amino acids with tRNA anticodons, dictating the assignment of codons to amino acids.

Because of their central role in protein synthesis, engineered and synthetic tRNAs emerged as essential components of orthogonal translation systems that are designed to incorporate non-canonical or even unnatural amino acids into proteins in cells and cell-free systems. Furthermore, recent efforts have used either normal wild-type or engineered tRNAs to correct genetic defects that cause human disease. Since 11% of inherited human genetic diseases are caused by premature stop codons, nonsense suppressor tRNAs are of increasing interest for applications in tRNA therapeutics. We recognize that both synthetic biology and therapeutic applications of tRNAs will rely on nonsense and in some cases missense suppressor tRNAs, to generate novel proteins or to correct genetic defects. Thus, this Research Topic for *Frontiers in Genetics* features genetic code expansion and studies probing the role of tRNAs in translation for applications in synthetic biology and medicine.

## Engineering of novel orthogonal pairs for genetic code expansion

**FIGURE 1:**
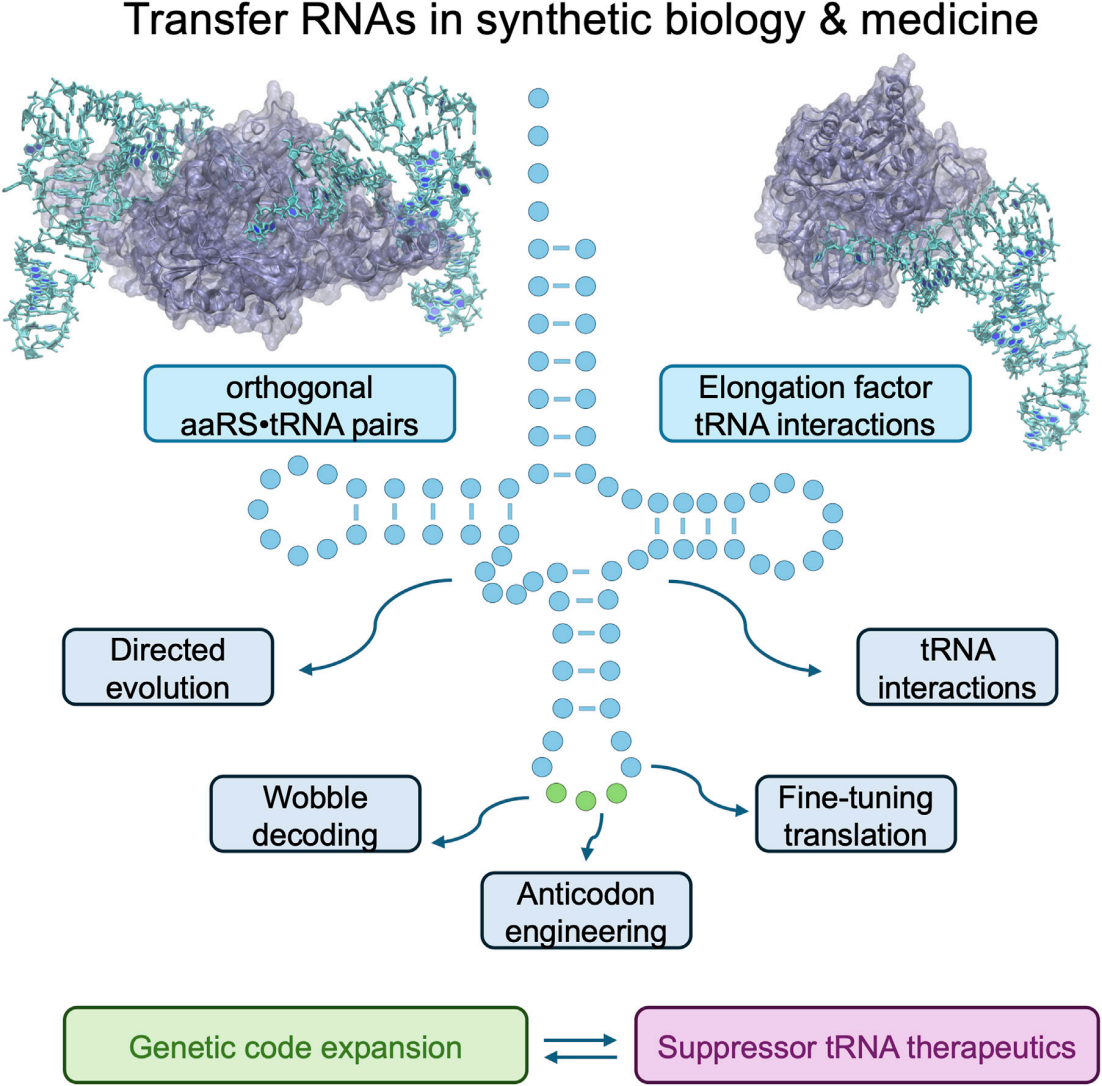
Transfer RNAs in synthetic biologly and medicine.

Several contributions in this Research Topic focus on the role and engineering of aminoacyl-tRNA synthetases and tRNAs in genetic code expansion (Figure 1). Research in this field has enabled the production of recombinant proteins with genetically encoded non-canonical amino acids, such as phosphoserine and *N*-acetyl-lysine. Jiang et al. present an engineered pyrrolysyl-tRNA synthetase (PylRS), which, rather than its natural substrate pyrrolysine (Pyl), instead directly charges tRNA^Pyl^ with a d-amino acid. Successful incorporation of d-phenylalanine analogs (DFAs) into both the superfolder green fluorescent protein and human heavy chain ferritin was demonstrated. Incorporation of d-amino acids is selected against in the endogenous translational machineries, where only l-amino acids are incorporated with high fidelity. The development of this newly evolved PylRS·tRNA^Pyl^ pair will allow the site-directed incorporation of d-amino acids into a diverse array of recombinant proteins. Potential future application areas include using genetically programmed d-amino acids to generate optically active proteins for therapeutic applications or the evolution of novel protein functions that are inaccessible to normal cells.

## Engineering principles for tRNAs to expand the genetic code

While significant progress has been made in engineering the aminoacyl-tRNA synthetases, such as PylRS, to expand the palette of different proteinogenic amino acids, the engineering of tRNAs has only more recently come into focus as a major approach to optimize and tune nonsense and missense suppression systems (Figure 1).

Kim et al. reviewed the engineering efforts of tRNAs to enhance unnatural amino acid incorporation efficiency, and to improve protein production with unnatural amino acids, and improve orthogonality to reduce interactions with endogenous aaRSs and tRNAs. The authors review principles of tRNA engineering including applications of directed evolution as well as rational design approaches to tRNA engineering. In addition, the authors analyze these different and complementary methods in the context of tRNA structural requirements needed to support tRNA aminoacylation and translational fidelity. Weiss et al. reviewed recent literature on tRNA engineering principles to tune translational fidelity, consolidating examples on tRNA engineering to allow for improved elongation factor TU (EF-Tu) binding, refining codon-anticodon interactions and facilitating peptide bond formation on the ribosome.

Recognizing the untapped potential in tRNA engineering, designing and improving orthogonal tRNAs was the focus of the research efforts in an original research article by Schmitt et al., who explored the translation activity of tRNAs with adenosine in the wobble position 34. Their overall goal was to use sense codons rather than stop codons, to expand the genetic code with non-canonical amino acids. Anticodons with an adenosine in the wobble positions are often modified to inosine, allowing for expanded decoding of multiple codons with the I34 containing tRNA to base pair with U, C, or A at the wobble position. Since this fact has important implications for the fidelity and specificity of sense codon reassignment, the authors evaluated the missense suppression efficiencies of 15 of the 16 possible orthogonal tRNAs with A34 anticodons and found that the I34 modification only occurred in a minority of the engineered tRNAs. Finally, Awawdeh et al. review the established and potential applications of suppressor tRNA as therapeutics in treating genetic diseases and highlighted how lessons learned from the field of genetic code expansion can be applied to further engineer suppressor tRNAs for medical applications.

In summary, the original research and review articles in this Research Topic highlighted approaches to tRNA and aminoacyl-tRNA synthetase engineering to expand the genetic code. Knowledge generated from decades of genetic code engineering will allow the development of new and improved tRNAs for both genetic code expansion as well as their novel application as therapeutics for genetic disease in humans.

